# A Systematic Review and Conceptual Framework of Biophilic Design Parameters in Clinical Environments

**DOI:** 10.1177/19375867221118675

**Published:** 2022-08-22

**Authors:** Bekir Huseyin Tekin, Rhiannon Corcoran, Rosa Urbano Gutiérrez

**Affiliations:** 1 School of Architecture, University of Liverpool, United Kingdom; 2 Department of Primary Care and Mental Health, Institute of Population Health, University of Liverpool, United Kingdom

**Keywords:** therapeutic environment, biophilic design, systematic review, healthcare settings

## Abstract

**Objectives:**

To provide a live-experience knowledge base about biophilic design parameters and environmental features to inform policy and design in clinical therapeutic environments.

**Background:**

It is increasingly important to review hospital design to make the best use of the affordances of natural elements in supporting both patients’ and staff’s physical and psychological well-being. The biophilic design theory provides an appropriate design approach. However, current biophilic design frameworks fail to provide efficiently standardized guidance. This systematic review aims to examine the experience of hospital users (patients and staff) with a view to informing a standardized biophilic design framework to improve future design in this context.

**Methods:**

This study performed a review and synthesis of nine studies identified using systematic procedures focusing on biophilic design features in healthcare environments.

**Results:**

The study identified a selection of biophilic parameters specifically relevant to this building typology, according to three different user groups: outpatients (fresh air, light-daylight, thermal comfort, welcoming and relaxing), inpatients (feeling relaxed and comfortable, prospect refuge, security and protection, light-daylight, view), and staff (privacy-refuge, quietness).

**Conclusions:**

The systematically identified studies helped to identify and rank the biophilic design parameters that appear the most critical for promoting and supporting human health and well-being in clinical therapeutic environments from the user’s perspective. It also provides an up-to-date compilation of crucial design interventions related to biophilic parameters and as such provides benchmark information for future research and design guidance in these environments.

## Introduction

Considerations about the quality of the environment in which healthcare is delivered can be tracked as early as ancient history. In Western culture, healthcare architecture evolved from the *Asclepeions* (healing temples) in Ancient Greece (Sternberg, 2009), to the military infirmaries in use during Roman times, *Valetudinariums* (Thompson & Goldin, 1975), and the hospitals run by the Church (monasteries) in Medieval and Renaissance times, which were later operated by town authorities during early Modernity (Verderber & Fine, 2000). These traditionally developed settings are early examples of biophilic thinking: They were usually built far from the high temperature, noise, dirt, and dust found in towns, and they typically offered a good view of nature and nearby freshwater sources. In the 18th century, hospitals started to diversify and specialize, producing medical research and training, laying the foundations of modern hospital care. The first design principles for hospital wards developed by Florence Nightingale in her 1863 book *Notes on Hospitals* were a crucial contribution toward establishing sanitation standards, which comprised considerations related to spatial layout, materials, and color, but most importantly to the quality of the environment, where natural elements such as daylight, fresh air ventilation, and heating played a key role (Nightingale, 1863; Verderber & Fine, 2000). From the time of this publication up to the Second World War, there was little literature on hospital design, however, soon after the war, the UK government started a proactive initiative for planning and postdesign evaluation of this complex and costly building typology, as part of the new vision for the modern city (Kenny & Canter, 1979; Stone, 1976). Post-war hospital planning privileged the building’s circulatory systems and mechanization with the aim of increasing efficiency in the use of human and technical resources, rationalizing and accelerating the delivery of clinical care (reducing inpatient lengths of stay to the minimum clinically necessary, and through increases in day surgery and outpatient treatment; Hughes, 1997). Nightingale’s principles were progressively disregarded in this process, which together with the dramatic growth of urbanization, the advent of the germ theory, and rapid changes in medical technology, led to an environmental approach to healthcare exclusively focused on healing through medical interventions (Murphy & Mansfield, 2017). From mid-20th century to today’s “mega hospitals” (Verderber & Fine, 2000), “mall hospitals” (Sloane & Sloane, 2002), or “factory-hospitals” (Jencks, 2017), healthcare environments have focused on the goals and objectives of the organization (fast physical recovery, mass health working like a machine), while neglecting the users (staff, patients) concerns and aspirations and, with this, their emotional, mental, and spiritual health (Abdelaal & Soebarto, 2019; Murphy & Mansfield, 2017; Silverstein, 2009). This is particularly important for patients who are diagnosed with a chronic condition and are undergoing treatment, as many studies have confirmed that they may experience high levels of psychological discomfort, with many experiencing fatigue, anxiety, or depression (Blazer et al., 1994; Guthrie, 1996; Mayou et al., 1991; McDaniel et al., 1995; Turner & Kelly, 2000; Zabora et al., 1997). There is research evidence that corroborates that the physical qualities of the setting in which a patient receives healthcare positively influence health outcomes in those mental disorders (Evans, 2003; Galea et al., 2005; Laursen et al., 2014; Moore et al., 2018; Rao et al., 2007; Ulrich et al., 1991; Yadav et al., 2018). In this context, it has become progressively important to review hospital design, and with it the concept of therapeutic environment, as it should not only be a place where patients are treated with the most advanced medicine and technology but also a place that supports their users (staff, and patients and their families), in psychological, emotional, and social terms (Smith & Watkins, 2016; Ulrich, 2008). Since the 1950s, research has been increasingly investigating optimal healing environments (e.g., stress recovery theory, attention restoration theory, therapeutic environment theory, salutogenesis, supportive design theory), bringing to the forefront the need to include other parameters in our design briefs, where the role of nature, and with it, the application of biophilic design, proves to be paramount.

Biophilic design as a discipline refers to an innate human connection to nature and natural processes, and the fulfilment of this connection promotes health and well-being in the environment we inhabit (Kellert et al., 2011; Kellert & Calabrese, 2015; Kellert & Wilson, 1993). Exposure to nature is associated with multiple health benefits (Kuo, 2015; Mcsweeney, 2014), including pain reduction, less medication, lower blood pressure, faster recoveries, and decreased all-cause mortality in general (Park & Mattson, 2009; Ulrich, 1984). Additionally, biophilic design can have a profound impact on supportive care, especially for patients suffering chronic diseases, who often experience psychological distress, fatigue, anxiety, or depression (Clarke & Currie, 2009; DeJean et al., 2013; Evans, 2003; Laursen et al., 2014; Moore et al., 2018; Turner & Kelly, 2000; Ulrich et al., 1991). It has also been shown that contact with nature promotes emotional, mental, and spiritual health; reducing stress; and triggering positive shifts in mood (Abdelaal & Soebarto, 2019; Berman et al., 2008; Bratman et al., 2012, 2021; Murphy & Mansfield, 2017; Silverstein, 2009). Some examples of effective biophilic healthcare environments within hospital settings are Alder Hey Children’s Hospital in Liverpool (designed by BDP in 2015), Circle Hospital in Bath (designed by Foster and Partners in 2009), or the Cancer Centre at Guy’s Hospital in London (designed by RSHP in 2016). There are also outstanding examples of biophilic healthcare environments within nonclinical settings (places where healthcare is delivered but not medical treatment), such as the Maggie’s Centers (Tekin et al., 2022). In all of these examples, the design focuses on human-centered environments, seeking to support physical and psychological well-being through close contact with nature, taking the users’ views into consideration, and showing as much care for humanity as efficiency.

The design parameters that relate to biophilia have been examined and categorized in three established general frameworks: (a) Kellert (Kellert et al., 2011) proposed a framework with 72 design parameters (usually termed as “patterns”) classed into six groups, (b) later on Kellert revised his study (Kellert & Calabrese, 2015) proposing a framework with 24 parameters organized in three groups, and (c) Terrapin Bright Green (Browning et al., 2014) proposed an alternative framework using 14 parameters also organized in three groups as shown in Table 1.

**Table 1.** Biophilic Design Parameters.

Framework	Groups	Parameters (‘patterns’)
Kellert & Calabrese, 2015	Direct Experience of Nature	• Light • Air • Water • Plants • Animals • Weather • Natural landscapes and ecosystems • Fire
	Indirect Experience of Nature	• Images of nature • Natural materials • Natural colors • Simulating natural light and air • Naturalistic shapes and forms • Evoking nature • Information richness • Age, change, and the patina of time • Natural geometries • Biomimicry
	Experience of Space and Place	• Prospect and refuge • Organized complexity • Integration of parts to wholes • Transitional spaces • Mobility and wayfinding • Cultural and ecological attachment to place
Browning et al., 2016	Nature in the Space	• Visual Connection with nature • Non-Visual connection with nature • Non-rhythmic sensory stimuli • Thermal & airflow variability • Presence of water • Dynamic & diffuse light • Connection with natural systems
	Natural Analogues	• Biomorphic forms & patterns • Material connection with nature • Complexity & order
	Nature of the Space	• Prospect • Refuge • Mystery • Risk/Peril

The scoping review of the literature by the authors confirms that there are three areas in which biophilic design has been developed: as investigated in research institutions, as used in design practice, and as established in building standards. Examining each of these areas uncovers several issues and disconnections. In scientific academic environments, there is abundant research on many of the different parameters of biophilic design, but this research examining the effects of nature on humans has happened separately for specific aspects of the design parameters and has not been brought together in a holistic and coherent way to support the frameworks. The design recommendations provided by the existing frameworks, the WELL Building Standard and the Living Building Challenge (certification schemes created to support the nourishment of wellness in the built environment; International Living Future Institute, 2019; International WELL Building Institute, 2020), are too broad and generic and developed from a Western perspective. These biophilic design frameworks don’t differentiate the level of value of each design parameter for each context and therefore, as design instruments, are too vague. Our position is that a biophilic design framework can only be efficient if it is specifically adapted to building function and geographical and cultural context. For instance, these frameworks recommend daylight as a parameter beneficial to humans but don’t specify adjustments regarding the daylighting requirements needed in a hospital of those needed in an educational building, or regarding the biological needs of people who live in extreme climates (e.g., northern latitudes or desert climates), or even regarding cultural dictates that might prioritize some parameters over biological needs (e.g., privacy over daylight in Muslim cultures). Therefore, to be able to provide efficient design guidance, it is necessary to determine a selective hierarchical structure for each context, as specific parameters from within the established general frameworks become especially relevant for the users.

***…a biophilic design framework can only be efficient if it is specifically adapted to building function and geographical and cultural context***.

***…to provide efficient design guidance, it is necessary to determine a selective hierarchical structure for each context, as specific parameters from within the established general frameworks become especially relevant for the users***

The goal of this study is to provide an account of the generated knowledge from users’ experiences in order to better inform human-centered policy and design by proposing a biophilic design framework specific to healthcare settings in the UK context. This article specifically discusses a systematic review conducted to identify, compare, and synthesize the published scholarly literature on biophilic design parameters and their impact on human health and well-being within clinical therapeutic environments from the user’s perspective.

## Method

This study followed a systematic review methodology with the aim to find all relevant published research that would answer the review question: *Which biophilic criteria are most critical in a clinical therapeutic environment and how do they inform design?*

The following criteria were decided for inclusion and exclusion of literature. The search was carried out in six different databases, spanning the period between 1973, when Fromm coined the term biophilia, and September 26, 2021. The language was limited exclusively to English. In order to avoid biased or less reliable data, the search was limited to peer-reviewed academic journal publications. The basic search syntax with Boolean operators was (“biophilic design” OR biophil* OR “natural design” OR “restorative design” OR “ecological design”) AND (“therapeutic environment” OR “healing environment” OR hospital OR healthcare OR “care center”) AND (well-being OR depres* OR anxi*). However, depending on the search limits of the used bibliographic databases, these codes showed variations.

**Figure 1.**
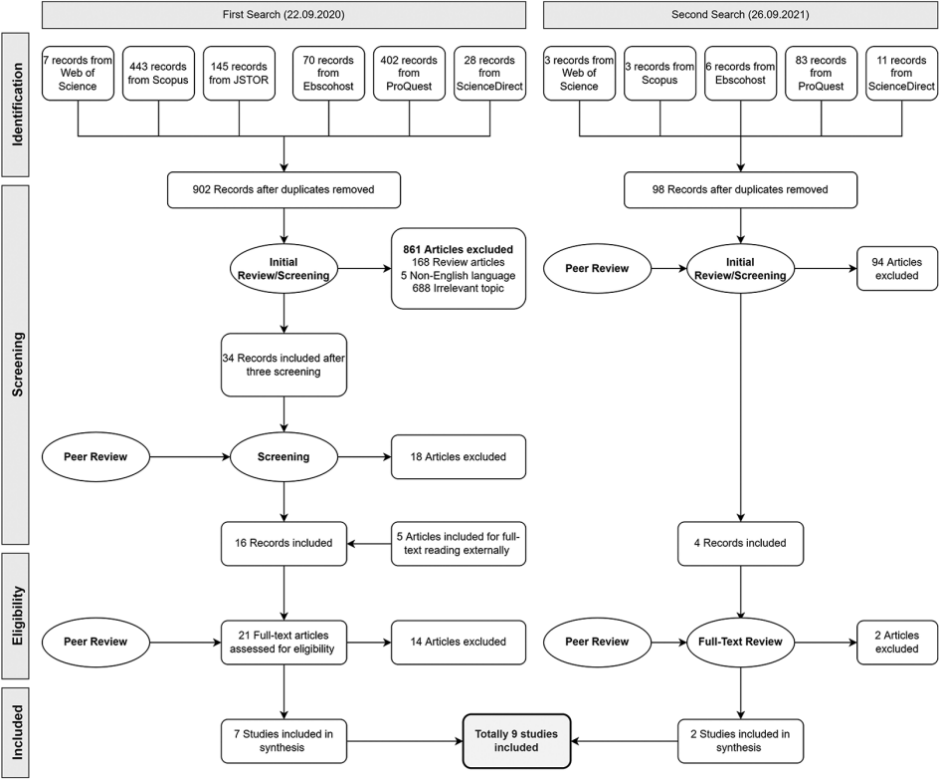
Identification of the included articles in the systematic review after applying the inclusion–exclusion criteria.

The search was concluded by exporting a total of 1,201 publications to Rayyan QCRI (https://rayyan.ai), a software that supports systematic review processes by expediting the initial screening of abstracts and titles using a semi-automated system (Ouzzani et al., 2016). After eliminating 201 duplicates, the initial screening was completed by reading abstracts and, in some cases, whole texts. To verify that all standards were met carefully, the initial screening was performed five times and peer-reviewed. Finally, 20 studies were included for full-text reading. During the full-text reading period, five more publications were added externally for full-text evaluation (Figure 1). To prevent selection bias, two of the authors read and reviewed the 25 full-text studies separately. Subsequently, nine papers were included in the synthesis (Abdelaal & Soebarto, 2019; Blaschke et al., 2017, 2018; Nejati et al., 2016; Peditto et al., 2020; Putrino et al., 2020; Tanja-Dijkstra & Andrade, 2018; Tinner et al., 2018; Wiltshire et al., 2020).

Table 2 shows the background information about the included studies. All nine studies were published between 2016 and 2020. Four of the studies were conducted in the United States, three in Australia, one in the United Kingdom, with one study co-conducted between the UK and the Netherlands. Five of the studies were published by health and/or medicine related authors, two studies were published by academics in environmental disciplines and two were conducted by academic architects. Additionally, the majority of the publications (seven) were focused on cancer care settings.

**Table 2.** Background Information of the Included Studies.

Study	Country	Year	Field
1	Australia	2017	Faculty of Medicine
2	United Kingdom	2020	Department for Health
3	Australia	2017	Faculty of Medicine
4	United States	2020	Department of Design and Environmental Analysis
5	United States	2018	Department of Forest and Natural Resources Management
6	United States	2020	Department of Rehabilitation and Human Performance
7	United States	2016	Department of Architecture
8	Australia	2019	Department of Architecture
9	Netherlands–United Kingdom	2018	Department of Clinical, Neuro, and Developmental Psychology

Since all the included studies employed different research methodologies, the analysis of the papers and data extraction for this systematic review followed individual procedures for each subject. Another reason for this was that the obtained data relevant to biophilic design parameters were found in a wide range of expressions. The general overview of the selected studies is shown in Table 3. Studies 1 and 2 were qualitative studies, and the data were extracted after a second analysis of the statements and facts reported in these studies, using NVivo Version 12 software (NVIVO, 2012). Quantitative data from various groups of participants were represented within Studies 3–6. Studies 7–9 used mixed methods. Data from either patients or staff were collected in all nine studies.

**Table 3.** General Overview of the Included Studies.

Study	Reference	Method	Participant Number	Population/Context	Contribution to the Systematic Review
1	Blaschke et al. (2018)	Qualitative	20	Patient/oncology	Inpatient/clinical
2	Wiltshire et al. (2020)	Qualitative	18	Cancer patients	Outpatient/clinical
3	Blaschke et al. (2017)	Quantitative	38	Experts/oncology	Inpatient/clinical
4	Peditto et al. (2020)	Quantitative	104	Young cancer patient facilities	Inpatient/clinical
5	Tinner et al. (2018)	Quantitative	72 Staff 62 Patient	Staff and patient/cancer center	Staff, outpatient/clinical
6	Putrino et al. (2020)	Quantitative	496	Frontline healthcare workers/COVID-19	Staff/clinical
7	Nejati et al. (2016)	Mixed	10 Interviews 993 Surveys	Professional nurses and healthcare workers	Staff/clinical
8	Abdelaal & Soebarto (2019)	Mixed-method review	NA	Patients	Inpatient and outpatient/clinical
9	Tanja-Dijkstra & Andrade (2018)	Review-mixed	Case 1: 62 survey	Cancer patients	Outpatient and inpatient/clinical

The quality assessment tool used in this systematic review was adapted from a study by Holloway Cripps (Holloway Cripps, 2016) and modified in accordance with Boland et al.’s (Boland et al., 2017) systematic review guidelines. It assesses reliability through a checklist of 13 questions. Studies 3, 5, and 7 were deemed high quality (highly reliable). A number of questions (one, three, one, and two respectively) were not satisfactorily answered by Studies 1, 2, 4, and 6. Accordingly, these four papers were given a good quality rating. Finally, Studies 8 and 9 were evaluated as poor quality.

## Synthesis of the Biophilic Design Parameters

**Figure 2.**
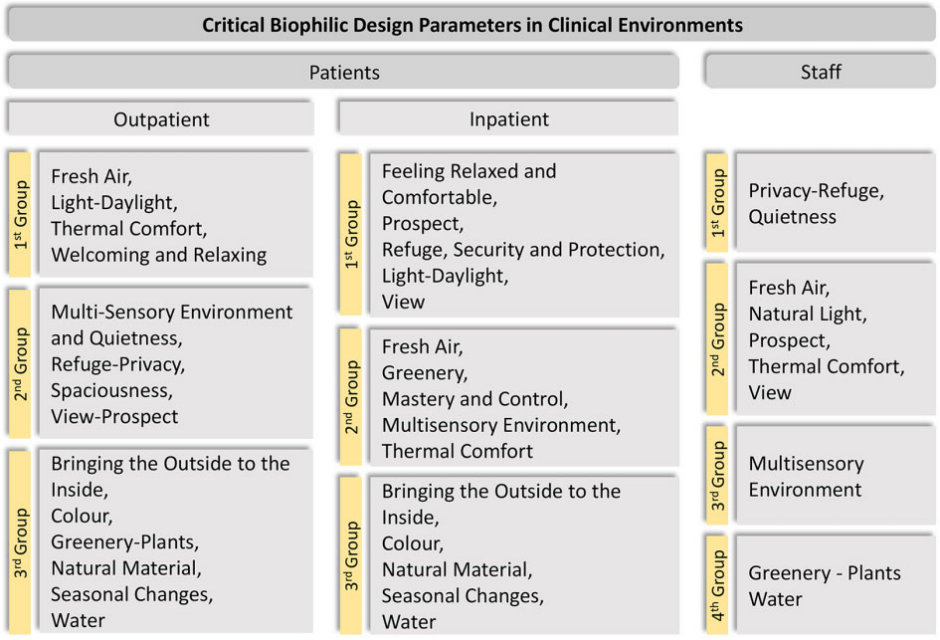
Classification by group of the identified biophilic design parameters in a clinical environment based on the review findings.

The first clear finding observed in the analysis of these studies was that healthcare environments cannot be evaluated as a single type of environment for all users in terms of their needs or for the significance of the biophilic design parameters. These clinical settings had to work both as therapeutic environments and as working environments for patients and staff respectively. Hence, the analysis examined biophilic design parameters from these two separate perspectives: patient-centered and staff-centered. The studies also revealed some differences in ambient perception between inpatient and outpatient users. Therefore, this distinction was also applied in the analysis. Figure 2 summarizes the classification of the identified biophilic design parameters in a clinical environment based on the review findings. The biophilic design parameters are organized according to the type of user and the level of importance, as certain parameters were reported to be more relevant to the respondents. As a result, up to four distinct groups were used to classify and rank these biophilic design principles: first group, second group, third group, and fourth group, in order of importance. The parameters contained within each group seemed to share the same level of importance and so were simply listed alphabetically. The following sections discuss how biophilic design can provide specific support to each of these users.

### Critical Biophilic Design Parameters for Patients

Studies 1–5, 8, and 9 investigated the clinical settings most regularly used by patients. Outpatient studies (Studies 2, 5, 8, and 9) focused mostly on specialist care units (e.g., chemotherapy), waiting rooms, and doctor rooms while the main emphasis of the inpatient investigations (Studies 1, 3, 4, 8, and 9) was wards and hospital rooms, and in some cases, outdoor areas.

Due to the use of different approaches and techniques across the studies, as well as the differences in the contextual environment of the respondents and the different scope in each case, the results were diverse, making the process of extracting general conclusions and ranking the discussed biophilic design parameters more challenging.

The outpatient column in Figure 2 summarizes the synthesized groups of essential biophilic design parameters for a clinical environment for these users. This ranking order reflects those biophilic design parameters that appear to most efficiently support stress-relieving, calming, comforting spaces in settings for outpatients. Five of the studies employed information relevant to inpatient environmental demands, particularly in cancer settings. Although the parameters found important for inpatient-based environments were not markedly different from those for outpatient-based environments, the identified priority differences may have an impact on environmental quality because the function of the spaces and the physical conditions of the patients differ. The inpatient column in Figure 2 summarizes the synthesized findings of what seem to be the most important biophilic design parameters for clinical environments for inpatient users. The most essential parameters for patients who spend most of their time in the wards or hospital rooms on their beds were view, prospect, and daylight through windows. These parameters were usually mentioned with regard to their visual impact as these patients’ mobility was limited. However, Studies 1, 3, and 4 clearly also stated the importance of accessible outdoor spaces, so those patients whose mobility is less restricted can be exposed to a multisensory environment. Refuge, security, and protection were other notable parameters in the top-ranked group, indicating the need to feel safe and secure due to their compromised health, and their high level of dependency on strangers and healthcare workers.

### Critical Biophilic Design Parameters for Staff

The research on staff (Studies 5–7) focused primarily on restorative characteristics of the environment. Studies 6 and 7 gathered information on staff break places, whereas Study 5 evaluated the clinical environment from the perspectives of both patients and staff. The staff column in Figure 2 summarizes the synthesized groups of biophilic design parameters for healthcare workers. The results for staff were synthesized into four different ranked groups, where the most important needs appeared to be Privacy and Refuge, with the need for Quietness mentioned frequently as well. Physical access to the outdoors was noteworthy in the investigations as well. Indoor greenery was unexpectedly low on the priority list. This seemed to be related to the added workload demands associated with their care and the possibility of harmful fungi presenting a risk of infection to patients. On the other hand, outdoor greenery within break areas was emphasized in the same investigations for its visual impact.

## Informing Design Practice

The previous section revealed the critical biophilic design parameters for healing environments. This section discusses how Studies 1–7 also informed how to implement these biophilic design parameters taking all the senses into consideration. The recommendations also refer to barriers in relation to the efficacy of biophilic design applications in practice.

According to Study 3, the most important barriers to creating a biophilic healing environment are generated in the decision-making process before designing the healthcare settings. Decision makers did not prioritize nature-based opportunities or “design thinking.” Clinical functionality, efficiency, cost restrictions, or habitual practice were often the main concern of healthcare facilities’ design regardless of the patients’ opinion and the quality of their experience. In order to eliminate these barriers, decision makers, designers, management, and administration must have knowledge about the importance of biophilic design so that decisions regarding site, layout, building orientation, surrounding views, and so on can be considered in the planning stage. Skilled professionals need also to consider the repair and maintenance needs of biophilic features within available maintenance budgets. Furthermore, the lack of knowledge and ability of the designers also leads to inappropriate design choices and executions, such as cold and stark spaces; too much hardscape, glaring materials or materials too hot to the touch; uncomfortable furniture; environments that are too demanding, complex, static, or under-stimulating; insufficient shading or lighting; and structures that cast odd shadows that could raise anxiety.

As the parameters changed to some extent depending on the user group, the suggestions for practice also showed some variations in different spaces. However, some recommendations were applicable to the spaces used by all user groups. For example, for all user groups, indoor design should maximize the use of natural materials, natural colors, fresh airflow, natural light, safety, and security (Study 1, Study 3, and Study 4). Windows in particular should afford views from clinical areas onto landscapes and the outside world, appropriate natural light exposure without glare, natural airflow, and safety (Study 1, Study 3). For all users, it appears crucial to protect them from overstimulation such as overpowering scents, noise, loud sounds, allergy-inducing and toxic plants, adverse comfort conditions, and high-low temperatures coming from overexposure to the sun or shade (Study 1, Study 3).

***...for all user groups, indoor design should maximize the use of natural materials, natural colors, fresh airflow, natural light, safety, and security***.

Creating a nonclinical feeling was a significant design driver for both patients and staff too. Study 5 recommended that medical equipment should be hidden from the eye where possible, for example, in common spaces and waiting rooms. Although the recommendation for furniture in the studies did not directly refer to biophilic design, furniture choice can support a nonclinical feeling and a homely, comfortable environment.

Physical access to outside and natural settings was another over-arching consideration for its restorative properties (Study 4, Study 7). Outdoor settings should have easy and effortless access for patients and staff. Adequate greenery and comfortable amenities where users can relax were welcomed. Shade and sunny areas should be balanced in order to provide spots for personalized comfort (Study 3) and porches, courtyards, patios, balconies, terraces, and gardens were mentioned as positive design features (Study 7). Physical exercise opportunities for both staff and patients could be offered in outdoor spaces and adapted to patients’ physical abilities. Stroll gardens, walking paths with points of interest and distance markers (plant species, medicinal plants), meandering trails, and resting points were all mentioned. In terms of staff exercise opportunities, nature walks, mindful walking, mobility and balance training, gardening tasks, assisted walking, and labyrinths were recommended (Study 3).

Although the three groups shared similarities in terms of desired parameters, we will see in the following sections that the affordances provided by them for each group should take different approaches.

### Applications Specific to Patients

Ease of movement was one of the most important features of the buildings for patients. As such, the maximization of accessibility and the removal of barriers were stressed. This included rapid and easy access between outdoor settings, foyer-waiting rooms, and treatment settings (Study 5) with safety considered as an over-arching priority in relation to movement. For example, the use of nonslip surface materials, smooth paved paths, ramps rather than steps and color contrasting curbing along pathways were mentioned. Barriers to be avoided included mention of heavy doors, narrow doorways and pathways (Study 3). In order to provide physical access to the outside, all barriers and thresholds should be removed for patients with automatic doors suggested improving ease of access (Study 3). Patients also suggested collaborating with volunteer services to provide opportunities for assisted walks outside the hospital building and visits to hospital gardens and courtyards, as well as contact with therapy animals (Study 1).

The material choice and heating system were another concern in terms of thermal comfort of the patients, as it was reported that the environment often tended to be over hot in hospitals. In Study 2, it was suggested that plastic materials be avoided as furniture options as it increases temperature perception. One patient said: “The seats make you very hot. That was one thing that we did [give feedback about] because they’re plastic” (Study 2, p. 4).

Study 5 claimed that providing optimum thermal comfort is challenging, often depending on individual differences and preferences. Therefore, a focus on the provision of personal control devices like warmed blankets or heated seating during the cold season and small fans during the hot season was recommended.

As the restrictions of existing building standards sometimes bar natural biophilic design features, the environment can be supported by artificial or electronic scenes and screens, plants or nature-related artworks. It was felt, within Study 2, that even if the artificial elements were not able to enrich a sensory environment, they could bring a visual focus for distraction: *“I quite like visual scenes of the environment—mountains and streams that sort of thing. That is pretty commonplace [in this hospital] and* it’s quite restful” (Study 2, p. 4). In addition, during clinical procedures, digital devices with interactive nature displays and sounds, such as virtual reality headsets, can be used to distract patients from unpleasant thoughts and reduce anxiety (Study 1). Patients in Study 2 referred to the importance of distraction provided by some artworks, such as a photography exhibition in the setting: “There are ones [artworks] that you just see and you forget what you’re here for” (Study 2, p. 4).

Participants of Study 2 criticized the sensory environment of the hospital environment referring to overall noisiness, noises of medical equipment, “buzzing” of alarms and “bleep” of the drips. The poor auditory experience caused by the machines was explained by a high-grade lymphoma patient:

…By the end of it, I found the whole place really irritating and upsetting. I didn’t really realize how much it was affecting me until on the odd occasion my wife came and met me and I was on edge. And the noise was one of the big things, especially when they’re busy and it’s endless. Bingbong, bing-bong, bing-bong, bing-bong. (Study 2, p. 4)

Patients were sensitive to the smell of the hospital since they commonly experienced anxiety and nausea. A patient claimed: “The last thing you want to be doing when you come in through this—especially when you’re feeling nauseous—is to go into a place that smells” (Study 2, p. 4).

#### Inpatient environment

Artificial biophilic interventions were more commonly considered in inpatient-related studies, although inauthenticity of nature-based design elements was claimed as a barrier to nature engagement such as fake plants, fake scents and tokenistic, and corporate design. Indoor planting can be incorporated with potted plants and green walls when appropriate and with caution. Nonetheless, plants are strictly prohibited in some clinics due to infection risk. Thus, high-quality artificial plants or scents can be used to incorporate biophilic elements to some extent (Study 1, Study 3). Furthermore, patients reported using technology to listen to natural sounds to help them sleep better. The ability to appropriately scale visual, auditory, and tactile intensities are one advantage of such technology-based interventions (Study 1).

The vast majority of the inpatients’ time during their stay is spent in wards or hospital rooms, usually physically dependent on beds. Therefore, windows should provide uninterrupted views, prospects, and sufficient natural light exposure to the bed, along with natural ventilation (Study 1, Study 3, and Study 4). Windows design should also pay attention to privacy, safety, and refuge by providing one-way views. Indoor seats and inpatient beds that are strategically located to maximize the use of natural window views can motivate patients to take advantage of these opportunities (Study 1, Study 3). In Study 2, the windows were criticized in this regard because they were too high to benefit from a direct line of sight to the outside area. A patient claimed, “you can’t even see a tree moving or anything” (Study 2, p. 4). Another participant expressed the general yearning of cancer patients for pleasant sights: “You just think ‘if only that were a nice garden space that you could wheel your drip out to and get a glimpse of cloud” (Study 2, p. 4).

Additionally, Study 4 recommended some environmental characteristics in indoor spaces that contribute to refuge, welcoming-relaxing feelings, and a sense of homeliness. Private bathrooms, private rooms, visitor beds in the patient rooms, family-patient or patient-only lounge, personal desks, closet spaces, and access to a kitchen were seen as important characteristics in this regard.

#### Outpatient environment

The entrance to the facility is an important space as arriving people often face high levels of stress and anxiety. It was felt that creating a welcoming atmosphere with biophilic touches can relax people. The use of natural materials such as timber, natural wall colors, fish tanks, and natural objects, that were kept at the reception and hospital foyer in Study 2, was well-received by patients, “because it’s very relaxing” (p. 4). As always, safety was emphasized, avoiding the inclusion of allergy-inducing elements, and slippery or otherwise challenging surfaces (Study 1, Study 2).

Spaciousness and calmness were sought in the clinical environment by patients, particularly in diagnosis rooms. Doors were especially mentioned in Study 2: sliding doors of a warm natural material as the best option. Furniture and materials should be comfortable and relaxing. High ceilings were recommended to create a spacious and well-lit environment, along with light accessible fittings which do not overwhelm patients. A participant shared her experience of receiving the diagnosis in Study 2 (p. 6):

The room is absolutely tiny. There’s not really anywhere to sit. And you have to actually sit on the bed. I sat on the bed, yeah. If you’ve got a visitor. Your visitor sits on the chair and then the doctor stands and talks to you. The image you get of being told you’ve got cancer is, you know, sitting in front of the desk, you know, the doctor sitting in a chair. He’s got reference books, you know. Whereas, you sort of feel like you’re sat in this little poky room because you’re not important enough. And you just feel that you…it’s just really awkward to be told you’ve got cancer sitting on a bed, tiny little room with two doctors standing there—because mine was two doctors standing—talking to you. So, for them to now tell me I am terminal, standing there in this tiny little room, no window at all…I wasn’t sitting comfortably, as I was sitting on the bed. They need to make you feel that they are talking to you and you alone, they’re not standing waiting to go out the door to talk to someone else.

The waiting rooms should allow sufficient daylight exposure, view, and fresh air and generate a quiet environment to reduce patients’ stress levels and the need to leave the room for relaxation (Study 2, Study 5). Easy and rapid access to the outdoor environment where the patients are able to hear when their name is announced or see a board showing the list of announced people was recommended (Study 2, p. 6):

From the patient perspective, being spatially confined to the waiting room prevents patients from accessing their preferred places within the hospital site. Participants talked of being “stuck there” for fear of missing their name being called and therefore being unable to “go outside to the courtyard” for its “scenery” and “fresh air.”

An efficient healthcare design should provide privacy as well as socializing opportunities (Study 3). Socializing opportunities can be created through spatial arrangement of seating and gathering options (Study 2), the inclusion of communal spaces, children’s play areas, semiprivate enclosures for personal conversations, and even BBQ areas (Study 3). However, socializing should not be enforced by the environment. Rather the environment should be flexible to accommodate socializing and withdrawal spaces (Study 2).

According to Study 5, as the most commonly preferred location within the treatment rooms, seats near to windows should be maximized, and the spatial arrangement should be designed to deliver optimum daylight and provide uninterrupted views for a larger portion of the room. In practice, an open-plan layout provides the highest exposure to daylight and socializing opportunities, but it also creates a noisier environment and impacts the provision of withdrawal spaces. Therefore, the inclusion of open-plan spaces needs more thought in order to create a balance of socializing and privacy and tranquility (Study 5).

Privacy can be provided through zoning or screening or by providing solitary spaces for rest or contemplation. A patient explained her experience after being diagnosed with cancer, in Study 2:

I remember going out and crying in the waiting room with my daughter. I was just hugging her. She was crying. She was crying and I was crying. And it was in front of everybody. We had nowhere to go that was a private space. (p. 5)

The spatial arrangement between waiting rooms and chemotherapy rooms should consider privacy, and patients mentioned that waiting areas in front of the toilets should be planned to also offer more privacy (Study 2).

### Applications for Staff

Views through windows were a frequently desired feature within staff indoor break areas (Study 7). Study 7 also revealed that visual or physical contact with the outside world and biophilic elements (i.e., view, prospect, daylight) played a critical role in staff’s restoration. The importance of this connection was stated by participants as follows:

When I had a window it made all the difference in the quality of my day, being able to look at out and see what was going on, I think the access to a view or to daylight and to the changing of the time of the day and the seasons is critical to the mental health and well-being of the staff. (Study 7, p. 22)

In terms of staff needs, easy access to private and quiet spaces, such as break rooms or outdoor settings shielded from inside views where they should also be able to enjoy adequate daylight and thermal comfort, were the most desired environmental features (Study 2, Study 5). Staff break areas should be located in ways that provide easy and rapid access back to patients and also to outdoor spaces which appears to be one of the most critical applications of biophilic design for staff well-being (Study 7). It was reported in Study 7 that participants should have access to rooftop gardens where they could have direct access to the nursing unit, and a patio garden directly accessible to the staff break rooms and cafeteria. The general idea of desirable indoor break area location followed the same concern, easy and rapid access to patients:

If they’re not able to have immediate access back to the unit, like if the break room is not on the unit, then oftentimes they won’t take breaks, You need to get away from the unit, at least behind a door so that the noise is not crazy and you’re not hearing everything. But that being said, you also can’t go very far away because your patients are sick and if you’re their nurse, it’s really difficult to not be right there. (Study 7, p. 29)

In fact, Study 7’s interview results reported that the most powerful stress reliever was the provision of direct access to the outdoors, because of the opportunities to direct contact with natural elements: “to walk in a garden, to be around diverse plants and flowers, to listen to the sound of water, and to receive direct sunlight” (p. 23). A participant described the ideal break area features for staff: “they had a beautiful staff lounge and it had a door that opens to a balcony, an outside balcony…just the ability to get fresh air, I think they would just love that” (p. 23).

***Staff break areas should be located in ways that provide easy and rapid access back to patients and also to outdoor spaces which appears to be one of the most critical applications of biophilic design for staff well-being***.

Staff specifically demanded a nonclinical homely environment in break areas where a sensorial connection with nature could provide a relaxing environment to reduce stress (Study 6). The furniture in break areas should be easily rearrangeable, and comfortable, for individual and group activities, with sofas and recliners, explicitly mentioned (Study 7).

Staff participants also recommended private outdoor break areas free from patients and their companions. Also, these outdoor areas should be enriched with greenery, trees, shade, tables, flowers, and water features:

In my perfect world, there would be plants—not anything too crazy that requires a lot of maintenance. There would be a water feature that just gave that noise, that waterfall noise, and then benches to sit on. It doesn’t have to be a big walking path because I just don’t have time…Trees, bushes, or flowers that have aroma to them; perhaps access to nature sounds [such as] running water or birds. I mean all of those elements of nature that we know nourish us as individuals. (Study 7, p. 23)

Privacy and being away from nonstaff sight were frequently emphasized:

If you’re going to have outdoor access, then I think it does need to be a quiet environment; again, private—it would be a private garden, not a garden like with families and kids running around, It has to be segregated because if families see staff members sitting outside…the family members are going to find them. (Study 7, p. 22)

**Figure 3.**
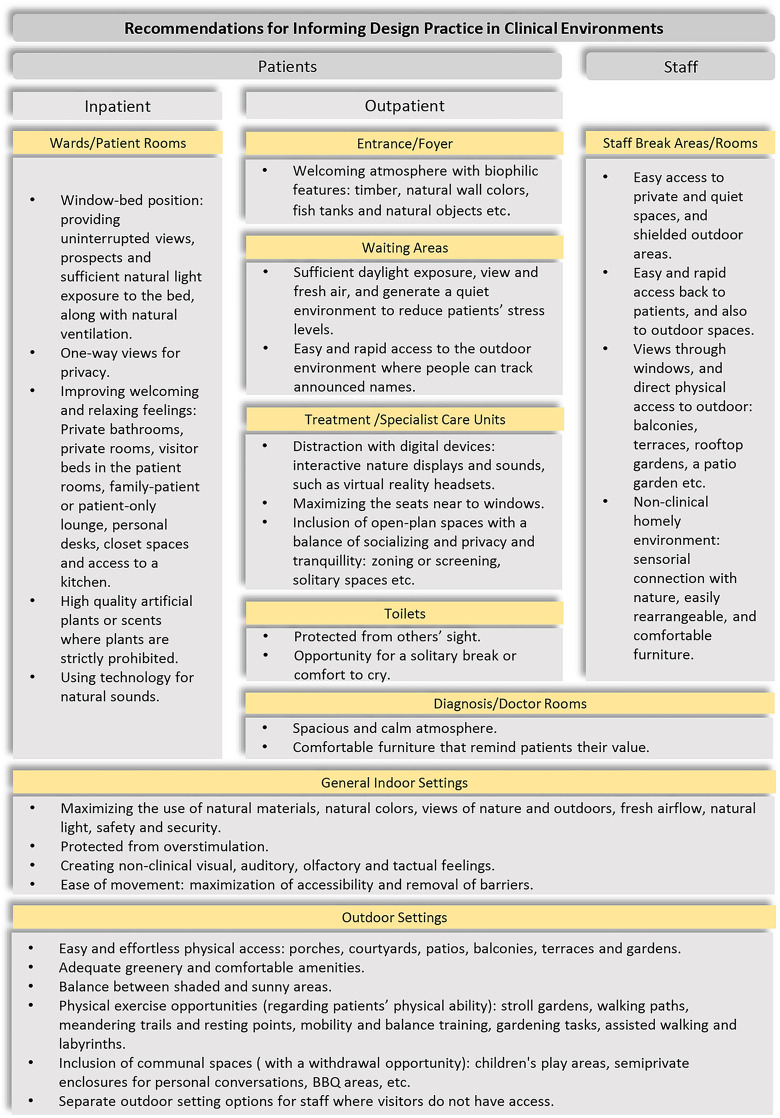
Summary of recommendations for informing design practice in clinical environments.

Figure 3 shows a summary of the main recommendations for each user group.

## Conclusion

In the context of the modern healthcare environments, it was confirmed that emotional, mental, and spiritual health issues are typically disregarded, while the main foci are physical treatment and cost. The environment has an influence on patients, particularly those affected by chronic illnesses as they visit hospitals regularly, as well as staff where under-resourced environments can give rise to health and well-being issues such as stress, depression, anxiety, and fatigue. The studies reviewed here focus on how the provision of biophilic design can mitigate these problems and promote a more welcoming and relaxing environment.

The synthesis of findings helped to identify and rank the biophilic design parameters that appear the most critical for promoting and supporting human health and well-being in clinical therapeutic environments, within and across three different user categories: outpatients (fresh air, light-daylight, thermal comfort, welcoming and relaxing), inpatients (feeling relaxed and comfortable, prospect refuge, security and protection, light-daylight, view), and staff (privacy-refuge, quietness). This review also showed that inpatient, outpatient, and staff users had similar desires but sometimes divergent priorities and requirements and that the provision of the same or similar biophilic elements to different groups could support distinct affordances. Therefore, future research should investigate the different building typologies and programs based on their specific user groups and contexts to provide efficient and rigorous biophilic design frameworks. This analysis also provides an up-to-date compilation of crucial design interventions related to biophilic parameters (summarized in Figure 3) and as such provides benchmark information for future research and design guidance in these environments.

The main limitation of this review was that not all the examined studies had as their main aim to produce data directly related to the assessment of biophilic design but rather to general hospital design environments. However, this could also be a benefit allowing a better understanding of the value of nature-based design where it fits within general healthcare design. It was also noticed that the available case studies were limited in number, however, they were systematically selected based on our criteria. This stringent selection is actually very important, because it frames the research to meet our specific goals and provides the necessary rigor to produce a substantive contribution to this early literature by revising it within this specific frame. The selected studies were localized in industrialized Western countries and typically of less than high methodological quality. Studies 8 and 9, showing the lowest level of reliability, proved not to contradict the high-quality studies but did not offer any further evidence to the synthesis, having no input into the design recommendations. Climate and culture influence human perceptions of nature, so as more research is conducted in various regions, climates, and cultures, a wider range of data will contribute toward more effective biophilic design frameworks.

A previous study by the authors focused on nonclinical therapeutic environments in the United Kingdom (Tekin et al., 2021, 2022), and identified other key biophilic design parameters, such as curiosity and sense of belonging, which were expected to be encountered in this review too. However, the discussed papers did not include any reference to them in the design of clinical environments. We suggest this should be further explored. Lastly, a rigorous design framework should be benchmarked against objective scientific data and qualitative primary data about the impact of biophilic design on humans. This analysis of each of the biophilic design parameters is currently being researched by the authors through a thorough literature review and a mix of qualitative methods, which will provide a holistic discussion and further complement guidance.

## Implications for Practice

- The provision of a clear, specific, and contextual use of biophilic design can more effectively inform design and research of therapeutic environments.
- The research shows that the effective use of biophilic design parameters and their corresponding design interventions depends on the needs of the user (inpatients, outpatients, and staff).
- The identification and ranking of biophilic design parameters relevant to healing environments in accordance with each type of user is a crucial step toward the effective use of natural elements for therapeutic purposes.
- A compilation of up-to-date design interventions and environmental features connected to biophilic design parameters is needed to inform a revised theoretical framework, policy and design guidelines.

## References

[bibr1-19375867221118675] Abdelaal M. S. Soebarto V. (2019). Biophilia and salutogenesis as restorative design approaches in healthcare architecture. Architectural Science Review, 62(3), 195–205. 10.1080/00038628.2019.1604313

[bibr2-19375867221118675] Berman M. G. Jonides J. Kaplan S. (2008). The cognitive benefits of interacting with nature. Psychological Science, 19(12), 1207–1212. 10.1111/j.1467-9280.2008.02225.x 19121124

[bibr3-19375867221118675] Blaschke S. O’Callaghan C. C. Schofield P. (2017). Nature-based care opportunities and barriers in oncology contexts: A modified international e-Delphi survey. BMJ Open, 7(10), e017456. 10.1136/BMJOPEN-2017-017456 PMC5652460 29042387

[bibr4-19375867221118675] Blaschke S. O’Callaghan C. C. Schofield P. (2018). Cancer patients’ recommendations for nature-based design and engagement in oncology contexts: Qualitative research. Health Environments Research & Design Journal, 11(2), 45–55. 10.1177/1937586717737813 29134826

[bibr5-19375867221118675] Blazer D. Kessler R. McGonagle K. Swartz M. S . (1994). The prevalence and distribution of major depression in a national community sample: The national comorbidity survey. The American Journal of Psychiatry, 151(7), 979–986. 8010383 10.1176/ajp.151.7.979

[bibr6-19375867221118675] Boland C. Dickson M. G. Angela R. (2017). Doing a systematic review: A student’s guide. SAGE.

[bibr7-19375867221118675] Bratman G. N. Hamilton J. P. Daily G. C. (2012). The impacts of nature experience on human cognitive function and mental health. Annals of the New York Academy of Sciences, 1249(1), 118–136. 10.1111/j.1749-6632.201106400.x 22320203

[bibr8-19375867221118675] Bratman G. N. Olvera-Alvarez H. A. Gross J. J. (2021). The affective benefits of nature exposure. Social and Personality Psychology Compass, 15(8), e12630. 10.1111/spc3.12630

[bibr9-19375867221118675] Browning W. Ryan C. Clancy J. (2014). 14 Patterns of biophilic design: Improving health & well-being in the built environment. Terrapin Bright Green, LLC, 1–60. 10.1016/j.yebeh.2008.04.024

[bibr10-19375867221118675] Canter D. Kenny C. (1979). Evaluating acute general hospitals. In Canter D. Canter S. (Eds.), Designing for therapeutic environments. A review of research. Wiley.

[bibr11-19375867221118675] Clarke D. M. Currie K. C. (2009). Depression, anxiety and their relationship with chronic diseases: A review of the epidemiology, risk and treatment evidence. Medical Journal of Australia, 190(7 SUPPL.), S54–S60. 10.5694/J.1326-5377.2009.TB02471.X 19351294

[bibr12-19375867221118675] DeJean D. Giacomini M. Vanstone M. Brundisini F. (2013). Patient experiences of depression and anxiety with chronic disease: A systematic review and qualitative meta-synthesis. Ontario Health Technology Assessment Series, 13(16), 1. https://www.ncbi.nlm.nih.gov/pmc/articles/PMC3817854/ PMC3817854 24228079

[bibr13-19375867221118675] Evans G. W. (2003). The built environment and mental health. Journal of Urban Health: Bulletin of the New York Academy of Medicine, 80(4), 137–146. 14709704 10.1093/jurban/jtg063 PMC3456225

[bibr14-19375867221118675] Galea S. Ahern J. Rudenstine S. Wallace Z. Vlahov D. (2005). Urban built environment and depression: A multilevel analysis. Journal of Epidemiology & Community Health, 59(10), 822–827. 10.1136/JECH.2005.033084 16166352 PMC1732922

[bibr15-19375867221118675] Guthrie E. (1996). Emotional disorder in chronic illness: Psychotherapeutic interventions. British Journal of Psychiatry, 168(MAR.), 265–273. 10.1192/BJP.168.3.265 8833678

[bibr16-19375867221118675] Holloway Cripps G. K . (2016). Too hot, too cold, and then just right: Balancing form and function in biophilic designed office workspaces. Doctoral dissertation, University of Maryland University College.

[bibr17-19375867221118675] Hughes J. (1997). Hospital-city. Architectural History, 40, 266–288

[bibr18-19375867221118675] International Living Future Institute. (2019). Living building challenge SM 4.0: A visionary path to a regenerative future. https://livingfuture.org/lbc/

[bibr19-19375867221118675] International WELL Building Institute. (2020). WELL building standard v2. https://v2.wellcertified.com/en

[bibr20-19375867221118675] Jencks C. (2017). Maggie’s architecture: The deep affinities between architecture and health. Architectural Design, 87(2), 66–75. 10.1002/ad.2154

[bibr21-19375867221118675] Kellert S. Calabrese E. (2015). The practice of biophilic design. www.biophilic-design.com

[bibr22-19375867221118675] Kellert S. Heerwagen J. Mador M . (2011). Biophilic design: The theory, science and practice of bringing buildings to life (pp. 3–15). John Wiley & Sons.

[bibr23-19375867221118675] Kellert S. Wilson E. O. (1993). The biophilia hypothesis. Island Press. https://liverpool.idm.oclc.org/login?url=https://search.ebscohost.com/login.aspx?direct=true&db=cat00003a&AN=lvp.b1667370&site=eds-live&scope=site

[bibr24-19375867221118675] Kuo M. (2015). How might contact with nature promote human health? Promising mechanisms and a possible central pathway. Frontiers in Psychology, 6, 1093. 10.3389/fpsyg.2015.01093 Link to access the article: 10.3389/fpsyg.2015.01093 Link to access the article: https://www.frontiersin.org/articles/10.3389/fpsyg.2015.01093/full 26379564 PMC4548093

[bibr25-19375867221118675] Laursen J. Danielsen A. Rosenberg J. (2014). Effects of environmental design on patient outcome: A systematic review. Health Environments Research & Design Journal, 7(4), 108–119. 10.1177/193758671400700410 25303431

[bibr26-19375867221118675] Mayou R. Hawton K. Feldman E. Ardern M. (1991). Psychiatric problems among medical admissions. International Journal of Psychiatry in Medicine, 21(1), 71–84. 10.2190/NDPB-YCW9-BETA-AYJE 2066259

[bibr27-19375867221118675] McDaniel J. S. Musselman D. L. Porter M. R. Reed D. A. Nemeroff C. B. (1995). Depression in patients with cancer: Diagnosis, biology, and treatment. Archives of General Psychiatry, 52(2), 89–99. 10.1001/ARCHPSYC.1995.03950140007002 7848055

[bibr28-19375867221118675] Mcsweeney J. Rainham D. Johnson S. A. Sherry S. B. Singleton J. (2015). Indoor nature exposure (INE): A health-promotion framework. Health Promotion International, 30(1), 126–139. 10.1093/heapro/dau081; https://pubmed.ncbi.nlm.nih.gov/25252597/ 25252597

[bibr29-19375867221118675] Moore T. H. M. Kesten J. M. López-López J. A. Ijaz S. McAleenan A. Richards A. Gray S. Savović J. Audrey S. (2018). The effects of changes to the built environment on the mental health and well-being of adults: Systematic review. Health & Place, 53, 237–257. 10.1016/J.HEALTHPLACE.2018.07.012 30196042

[bibr30-19375867221118675] Murphy M. Mansfield J. (2017). Can architecture heal? Building as instruments of health. Architectural Design, 87(2), 82–89. 10.1002/ad.2156

[bibr31-19375867221118675] Nejati A. Shepley M. Rodiek S. Lee C. Varni J. (2016). Restorative design features for hospital staff break areas: A multi-method study. Health Environments Research & Design Journal, 9(2), 16–35. 10.1177/1937586715592632 26163571

[bibr32-19375867221118675] Nightingale F. (1863). Notes on hospitals. Longman, Green, Longman, Roberts, and Green.

[bibr33-19375867221118675] NVIVO, Q. (2012). Nvivo qualitative data analysis software. https://www.qsrinternational.com/nvivo-qualitative-data-analysis-software/home

[bibr34-19375867221118675] Ouzzani M. Hammady H. Fedorowicz Z. Elmagarmid A. (2016). Rayyan—A web and mobile app for systematic reviews. Systematic Reviews, 5(1), 1–10. 10.1186/s13643-016-0384-4 27919275 PMC5139140

[bibr35-19375867221118675] Park S. H. Mattson R. H. (2009). Ornamental indoor plants in hospital rooms enhanced health outcomes of patients recovering from surgery. The Journal of Alternative and Complementary Medicine, 15(9), 975–980. https://doi.org/Home.Liebertpub.Com/Acm, 10.1089/ACM.2009.0075 19715461

[bibr36-19375867221118675] Peditto K. Shepley M. Sachs N. Mendle J. Burrow A. (2020). Inadequacy and impact of facility design for adolescents and young adults with cancer. Journal of Environmental Psychology, 69(September 2019), 101418. 10.1016/j.jenvp.2020.101418

[bibr37-19375867221118675] Putrino D. Ripp J. Herrera J. E. Cortes M. Kellner C. Rizk D. Dams-O’Connor K. (2020). Multisensory, nature-inspired recharge rooms yield short-term reductions in perceived stress among frontline healthcare workers. Frontiers in Psychology, 11. 10.3389/fpsyg.2020.560833 PMC7714047 33329188

[bibr38-19375867221118675] Rao M. Prasad S. Adshead F. Tissera H. (2007). The built environment and health. The Lancet, 370(9593), 1111–1113. 10.1016/S0140 17868821

[bibr39-19375867221118675] Silverstein A . (2009). A history of immunology. Academic Press.

[bibr40-19375867221118675] Sloane D. C. Sloane B. C. (2002). Medicine moves to the mall. Johns Hopkins University Press. http://ebookcentral.proquest.com/lib/liverpool/detail.action?docID=3318068

[bibr55-19375867221118675] Smith R. Watkins N. (2016). “Therapeutic Environments”. From the Therapeutic Environments Forum, AIA Academy of Architecture for Health, WBDG – Whole Building Design Guide, accessed December 2021: https://www.wbdg.org/resources/therapeutic-environments+

[bibr41-19375867221118675] Sternberg E. M. (2009). Healing spaces: The science of place and well-being. Harvard University Press. https://books.google.com/books/about/Healing_Spaces.html?hl=tr&id=xyDqRAAACAAJ

[bibr42-19375867221118675] Stone P . (1976). Hospitals: The heroic years. Architects’ Journal, 164(50), 1121–1148.

[bibr43-19375867221118675] Tanja-Dijkstra K. Andrade C. C. (2018). 12—Healthcare settings. In Devlin A. S. (Ed.), Environmental psychology and human well-being (pp. 313–334). Academic Press. 10.1016/B978-0-12-811481-0.00012-3

[bibr44-19375867221118675] Tekin B. H. Corcoran R. Urbano Gutiérrez R. (2021). A new reading of therapeutic environments: Biophilic elements in Maggie’s Centres [Conference session]. The 8th International Conference on Architecture and Built Environment with AWARDs (pp. 289–298). www.s-arch.net

[bibr45-19375867221118675] Tekin B. H. Corcoran R. Urbano Gutiérrez R. (2022). The impact of biophilic design in Maggie’s Centres: A meta-synthesis analysis. Frontiers of Architectural Research. 10.1016/J.FOAR.2022.06.013

[bibr46-19375867221118675] Thompson J. D. Goldin G . (1975). The hospital: A social and architectural history (pp. 1975–349). Yale University Press. https://books.google.com/books/about/The_Hospital.html?hl=tr&id=POJzQgAACAAJ

[bibr47-19375867221118675] Tinner M. Crovella P. Rosenbaum P. F. (2018). Perceived importance of wellness features at a cancer center: Patient and staff perspectives. Health Environments Research & Design Journal, 11(3), 80–93. 10.1177/1937586718758446 29488391

[bibr48-19375867221118675] Turner J. Kelly B. (2000). Emotional dimensions of chronic disease. Western Journal of Medicine, 172(2), 124. 10.1136/EWJM.172.2.124 10693376 PMC1070773

[bibr57-19375867221118675] Ulrich R. S. (1984). View through a window may influence recovery from surgery. Science, 224(4647), 420–421. 10.1126/SCIENCE.6143402 6143402

[bibr49-19375867221118675] Ulrich R. S. Simons R. F. Losito B. D. Fiorito E. Miles M. A. Zelson M. (1991). Stress recovery during exposure to natural and urban environments. Journal of Environmental Psychology, 11(3), 201–230. 10.1016/S0272-4944(05)80184-7

[bibr50-19375867221118675] Ulrich R. S. Zimring C. Zhu X. DuBose J. Seo H. B. Choi Y. S. Quan X. Joseph A. (2008). A review of the research literature on evidence-based healthcare design. Health Environments Research & Design Journal, 1(3), 61–125. 10.1177/193758670800100306; https://pubmed.ncbi.nlm.nih.gov/21161908/ 21161908

[bibr51-19375867221118675] Verderber S. Fine D. J . (2000). Healthcare architecture in an era of radical transformation. Yale University Press. https://liverpool.idm.oclc.org/login?url=https://search.ebscohost.com/login.aspx?direct=true&db=cat00003a&AN=lvp.b2806681&site=eds-live&scope=site

[bibr52-19375867221118675] Wiltshire G. Pullen E. Brown F. F. Osborn M. Wexler S. Beresford M. Tooley M. Turner J. E. (2020). The experiences of cancer patients within the material hospital environment: Three ways that materiality is affective. Social Science and Medicine, 264(September), 113402. 10.1016/j.socscimed.2020.113402 33010714

[bibr53-19375867221118675] Yadav M. Chaspari T. Kim J. Ahn C. R. (2018). Capturing and quantifying emotional distress in the built environment. In Proceedings of the Human-Habitat for Health (H3): Human-Habitat Multimodal Interaction for Promoting Health and Well-Being in the Internet of Things Era—20th ACM International Conference on Multimodal Interaction, ICMI 2018. 10.1145/3279963.3279967

[bibr54-19375867221118675] Zabora J. R. Blanchard C. G. Smith E. D. Roberts C. S. Glajchen M. Sharp J. W. BrintzenhofeSzoc K. M. Locher J. W. Carr E. W. Best-Castner S. Smith P. M. Dozier-Hall D. Polinsky M. L. Hedlund S. C. (1997). Prevalence of psychological distress among cancer patients across the disease continuum. Journal of Psychosocial Oncology, 15(2), 73–87. 10.1300/J077V15N02_05

